# Structural Study of Europium Doped Gadolinium Polyphosphates LiGd(PO_3_)_4_ and Its Effect on Their Spectroscopic, Thermal, Magnetic, and Optical Properties

**DOI:** 10.1155/2018/4371064

**Published:** 2018-06-19

**Authors:** Saoussen Hammami, Nassira Chniba Boudjada, Adel Megriche

**Affiliations:** ^1^Université de Tunis El Manar, Faculté des Sciences de Tunis, UR11ES18 Unité de recherche de Chimie Minérale Appliquée. Campus Universitaire Farhat Hached, 2092 Manar 1. Tunis, Tunisia; ^2^Institut Néel, BP 166, 38042 Grenoble Cedex 9, France

## Abstract

Alkali metal-rare earth polyphosphates LiGd_(1-x)_Eu_x_(PO_3_)_4_ (LGP:Eu^3+^) (where x= 0, 0.02 and 0.04) were synthesized by solid-state reaction. The Rietveld refinement showed the following cell parameters: I 2/a space group, a=9.635(3) Å, b=7.035(3) Å, c=13.191(3) Å, *β*=90.082°, V= 894.214Å3, and Z=4. The similarity between R_F_=4.21% and R_B_=4.31% indicated that the realized refinement is reliable. The crystal structure consists of infinite zig-zag chains of (PO_4_)^3-^ tetrahedra, linked by bridging oxygen. The acyclic structure of polyphosphates is confirmed by infrared and Raman (IR) spectroscopies. A good thermal stability up to 940°C and paramagnetic behavior of these compounds were also proved by thermal analyses and magnetic susceptibility measurements, respectively. Excitation spectra revealed the charge transfer phenomenon between O^2-^ and Eu^3+^ (CTB), the energy transfer from Gd^3+^ to Eu^3+^, and the intrinsic 4f-4f transitions of Eu^3+^ where the electronic transitions were also identified. Moreover, LGP:Eu^3+^ can emit intense reddish orange light under excitation at 394 nm. The strongest tow at 578 and 601 nm can be attributed to the transitions from excited state ^5^D_0_ to ground states ^7^F_1_ and ^7^F_2_, respectively.

## 1. Introduction

Condensed alkali metal-rare earth polyphosphates, with the general formula M^I^RE^III^(PO_3_)_4_ (where M^I^= are alkali metal and RE^III^= are rare earth ions), have been extensively investigated thanks to their structural diversity [1–4] and their interesting magnetic [5], optic [6], and electric [7] proprieties. These polyphosphates are generally stable in normal conditions of temperature and humidity [8], which makes them useful for industrial applications. For example, Yamada et al. have used the LiNd(PO_3_)_4_ polyphosphate as a solid-state laser material [9, 10]. However, Z. Mua et al. have used LiEu(PO_3_)_4_ compound for white light-emitting diodes [11]. They are also used as promising scintillation material such as the Ce^3+^ doped MGd(PO_3_)_4_ compound [12].

In order to enhance the optical properties of polyphosphates, researches were oriented to doping them with metal-rare earths. In recent years, a great interest was accorded to europium earth-rare due to its outstanding photoluminescence feature [13–19]. In fact, the presence of well-defined energy levels in europium allows the emission of monochromatic and coherent radiations in solid laser.

The Gd–Eu couple is well known for its efficient conversion of the absorbed high-energy photons into two visible ones [20–24]. This phenomenon may be followed by a sequence of two steps of energies transfer. The first step is the transition ^6^G_J_→^6^P_J_ of Gd^3+^, involving the ^5^D_0_→^7^F_1_ transition of the Eu^3+^ ion. The one red photon related to the Eu^3+^  ^5^D_0_ → ^7^F_J_ transition is created. In the second step, the energy of Gd^3+^ is related to ^6^P_7/2_→ ^8^S transition which is transported over the Gd^3+^ sublattice and then transferred to another Eu^3+^ ion. This phenomenon leads to ^5^D_J_ emission (J = 0, 1, 2, or 3) [25].

This work describes the synthesis of LiGd_(1-x)_Eu_x_(PO_3_)_4_ polyphosphate, doped with different low percentages of europium (2 and 4%). The structural study of all obtained compounds is carried out with XRD diffraction. The infrared and Raman spectroscopies and magnetic and thermal analyses were recorded at room temperature. Moreover, the optical study through excitation and emission of Eu^3+^ ions spectra was also undertaken.

## 2. Experimental

The condensed phosphates LiGd_(1-x)_Eu_x_(PO_3_)_4_ (where x= 0, 0.02 and 0.04) were synthesized by solid-state reaction (methods of Hammami et al. 2017) [26]. A mixture of the reagents, Li_2_CO_3_, Gd_2_O_3_, Eu_2_O_3_, and NH_4_H_2_PO_4_, was prepared with the molar ratio (2.1:1:8) of Li:Gd:P, respectively. First, the raw materials were grounding in an agate mortar for one hour at least to homogenize the solid phase and improve the interatomic diffusion. Second, the mixture was introduced into the oven and submitted to the following thermal program. The first level was at 430°C to eliminate H_2_O, NH_3_, and CO_2_, the second one was at 730°C to get LiGd_(1-x)_Eu_x_(PO_3_)_4_ pure phase. Then, the obtained products were cooled with the rate of 2°C/min to ensure a better crystallinity. Finally, the synthesized polyphosphates were washed with boiling water and nitric acid solution (1mol/L) to eliminate the residual raw materials from the final product.

The proposed chemical reaction for polyphosphate synthesis is

(1) 8NH4H2PO4+1-xGd2O3+xEu2O3+Li2CO3⟶2 LiGd1-xEuxPO34+8NH3↑+12H2O+CO2↑

Samples were characterized using an INEL XRG 3000 (D5000T) diffractometer with monochromatic Cu K_*α*_ radiation. The diffraction pattern was recorded under 300K over the angular range 10-90° (2*θ*). The luminescence spectra were performed under ambient atmosphere via Xenius (the fluorescence Genius) spectrophotometer, at 591nm and 394nm for excitation and emission, respectively. The infrared spectra were recorded in the range of 250–1500 cm^−1^  with a Thermo Scientific Nicolet N10 MX using sample dispersed in KBr pellets. Raman analysis was carried out at room temperature, with 514.5 nm radiation from an argon ion laser as the excitation beam. A microscope allowed a selection of high optical quality regions in the crystalline sample. Thermal stability of Eu^3+^ doped LGP was measured with differential thermal analysis SETARAM TAG 16 operating from room temperature up to 1000°C with heating rate of 5°C min^−1^. Magnetic measurements were carried out using Quantum Design MPMSXL magnetometer with detection SQUID (at institute NEEL France).

## 3. Results and Discussion

### 3.1. Rietveld Refinement Data Analyses

**Figure 1:**
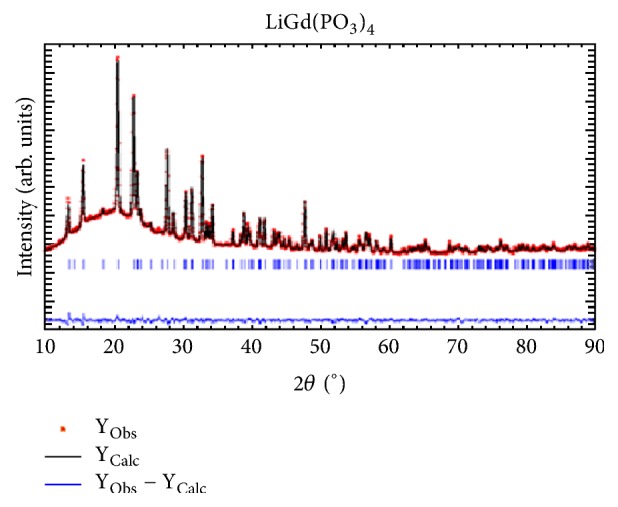
The Rietveld analysis of X-ray diffraction patterns for LiGd(PO_3_)_4_.

The Rietveld refinement of X-ray diffraction patterns of synthesized LiGd(PO_3_)_4_ samples is shown in Figure 1. The graph presents the experimental and the calculated data as well as the difference between them. As it is shown, the presence of only single phase was checked by Rietveld fitting quality through the reliability R factors: R_exp_, R_brag_, profile R_p_, and weighted profile R_wp_, which should be less than 10%. The final R factors, atomic coordinates, site occupancy, thermal displacement parameters, and their estimated standard deviations in parentheses for LGP are shown in Table 1. Interatomic bond distances and angles are given in Table 2. The new lattice parameters, derived from the Rietveld refinement, are a=9.635(3) Å, b= 7.035(3) Å, c= 13.191(3) Å, and *β*= 90.082° and with monoclinic space group I 2/a.

**Figure 2:**
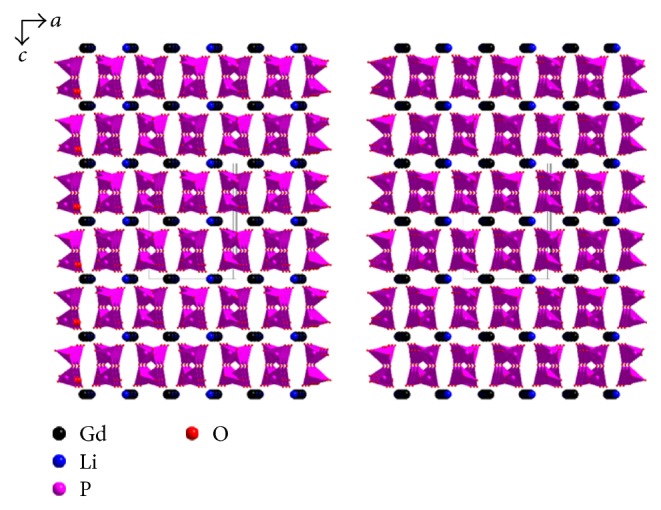
The structural arrangement of the LiGd(PO_3_)_4_ viewed in the (0 1 0) plane.

The LGP structure can be simply described as three-dimensional framework of GdO_8_ polyhedra linked to (PO_4_)^3–^ rings by Gd-O-P bridges. This framework delimits interesting tunnels with Li^+^ ions which are bonded to four oxygen atoms (LiO_4_). Each LiO_4_ tetrahedron shares all four O atoms with two LaO_8_ polyhedra and four different PO_4_ tetrahedra. A view of this structure projected along the b axis is shown in Figure 2.

### 3.2. X-Ray Powder Diffraction

**Figure 3:**
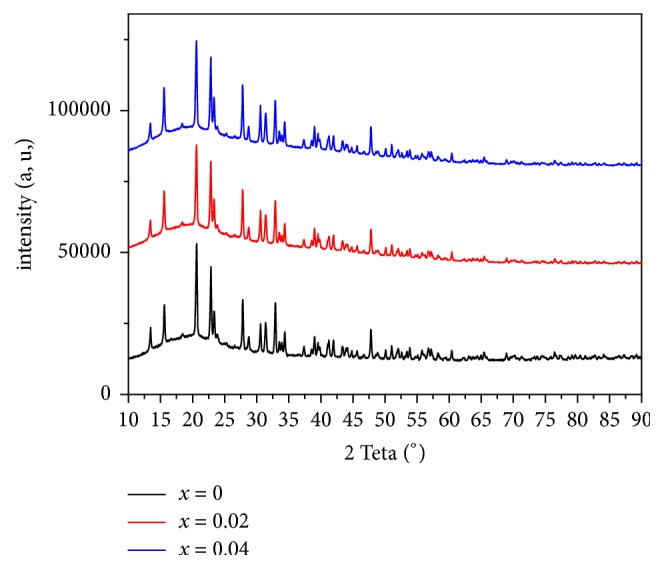
XRD patterns of LiGd_(1-x)_Eu_x_(PO_3_)_4_ ( x=0, 0.02 and 0.04).

X-ray diffraction patterns of LiGd_(1-x)_Eu_x_(PO_3_)_4_ (where x= 0, 0.02 and 0.04) are shown in Figure 3. The obtained crystalline phases are isotypes of the mother-phase LiGd(PO_3_)_4_ [27]. Mainly, it is shown that XR diffraction peaks of studied solids, with different percentages of europium, are described in a cell with a super space group I 2/a instead of C 2/c usually used in crystallographic data of the old LGP. The same XRD pattern is obtained for almost of synthesized compounds, even at high Eu^3+^ concentrations. However, a shift of the diffraction peaks to the lower 2*θ* is observed. This shift can be explained by Bragg's theory “**n*λ*= **2**d**_**hkl**_**sin*θ*”** (where *λ* is the X-ray wavelength (Cu K_*α*_ =1.5406Å), *θ* is diffraction angle, and d is interplanar distance of corresponding diffraction peaks). Therefore, *λ* is constant; it can be concluded that this shift is due to the increase of interplanar distance “d”. Considering the characteristics of Gd and Eu (ionic radius of Gd^+3^: 1.05 Å, Eu^+3^: 1.07 Å and atomic volume Gd: 19.9 cm^3^/mol, Eu: 28.9 cm^3^/mol), this phenomenon can be attributed to the distortion of the tetrahedra of polyphosphates upon europium insertion [28].

The crystallite size of obtained polyphosphates is calculated using Sherrer's equation below [29] and values are summarized in Table 3. Results show that the range of calculated crystallite size is between 42.49 and 42.79 nm, which prove that the synthesized compounds are nanometric.

(2) $$\begin{array}{c} \text{Sherre'r equation:}\;D=\frac{0.9\lambda }{\beta \cos\theta } \end{array}$$

where *λ* is the X-ray wavelength (Cu K_*α*_ =1.5406Å), *θ* is the Bragg diffraction angle, and *β*e is the full width at half -maximum (FWHM) in radian of the main peak of each XRD pattern.

### 3.3. Infrared and Raman Spectroscopy Investigations

#### 3.3.1. Infrared

**Figure 4:**
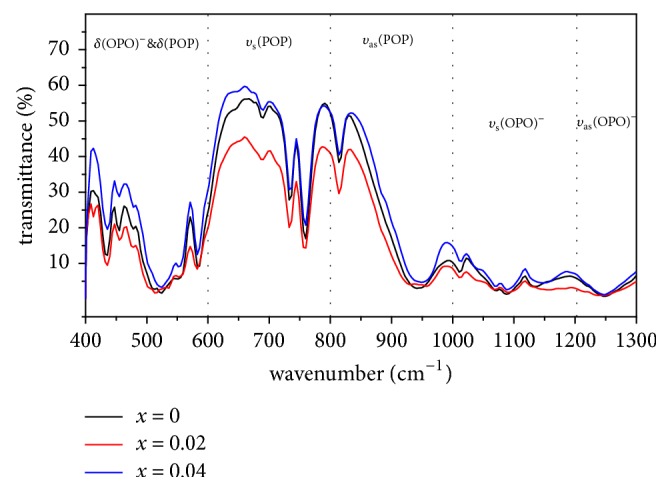
IR spectra of LiGd_(1-x)_Eu_x_(PO_3_)_4_ ( x=0, 0.02 and 0.04).

Figure 4 shows the IR spectra of all studied compounds. The comparison of spectra (Figure 4) and those obtained in previous works in literature for condensed polyphosphates [30, 31] proves that positions of infrared absorption bands are characteristic of phosphates with chain structures.

IR bands attribution is carried out based on (O-P-O)^−^ groups and P-O-P bridges vibrations [32, 33]. The IR absorption spectra show the presence of two bands around 1249 cm^−1^  which are assigned to the asymmetric stretching vibration (*υ*_as_) of O-P-O. The weak band observed between 1071 and 1136 cm^−1^  is attributed to the symmetric stretching vibration *υ*_s_ of O-P-O. The large and intense band around 944 cm^−1^  is assigned to the asymmetric vibration *υ*_as_ of P-O-P. We can also attribute the few bands at 689-818 cm^−1^  to the symmetric vibration *υ*_s_ (P-O-P). At low frequencies region, below 600 cm^−1^ , it is very difficult to distinguish the symmetric and antisymmetric bending modes of the (O-P-O) and (P-O-P) groups. The frequencies of the corresponding bands are given in Table 4.

The major difference between the IR spectra of cyclic polyphosphate and polyphosphate is the absence of vibration bands between 750 and 1000 cm^−1^ . In this range, IR spectroscopy confirms the structure as long as polyphosphates chains.

#### 3.3.2. Raman

**Figure 5:**
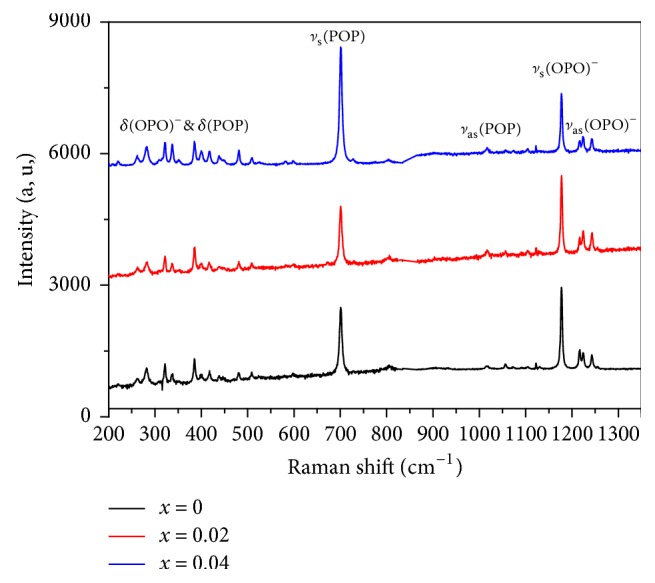
Raman spectra of LiGd(PO_3_)_4_ (x=0, 0.02 and 0.04) at 300 K.

The Raman spectra of LiGd_(1-x)_Eu_x_(PO_3_)_4_ (where x= 0, 0.02 and 0.04) at room temperature are shown in Figure 5. These spectra show the presence of many bands; the first intense band at 1178 cm^−1^  and the second at 700 cm^−1^  are assigned to antisymmetric stretching vibration mode *υ*_as_ (O-P-O) and symmetric stretching vibrations mode *υ*_s_ (P-O-P), respectively. The *υ*_as_ (P-O-P) asymmetric and *υ*_s_ (O-P-O) symmetric stretching vibration modes, respectively, appear in the 1000-1100 cm^−1^  and 1212-1296 cm^−1^  ranges. The bands under 599 cm^−1^  are attributed to the symmetric and the asymmetric bending vibrations (*δ*_as_ and *δ*_s_) of (O-P-O)^−^ and (P-O-P). The intense symmetric stretching vibrations bands around 700 and 1178 cm^−1^  are characteristic of phosphoric anions (PO_3_)_4_^4−^ [34].

Distinguishing characteristic cyclotetraphosphates and polyphosphates compounds exit also in the Raman spectrum. The symmetric stretching vibration of the P-O-P (*υ*_s_ (P-O-P)) has a single band at 700 cm^−1^ . This is the strongest of all the Raman vibration bands. That is because of the monoclinic symmetry of LGP doped Eu and the different positions of the lanthanide and alkali ions. The results of Raman spectroscopy can identify the structure of alkali metal lanthanide polyphosphates.

### 3.4. DTA (Differential Thermal Analysis)

**Figure 6:**
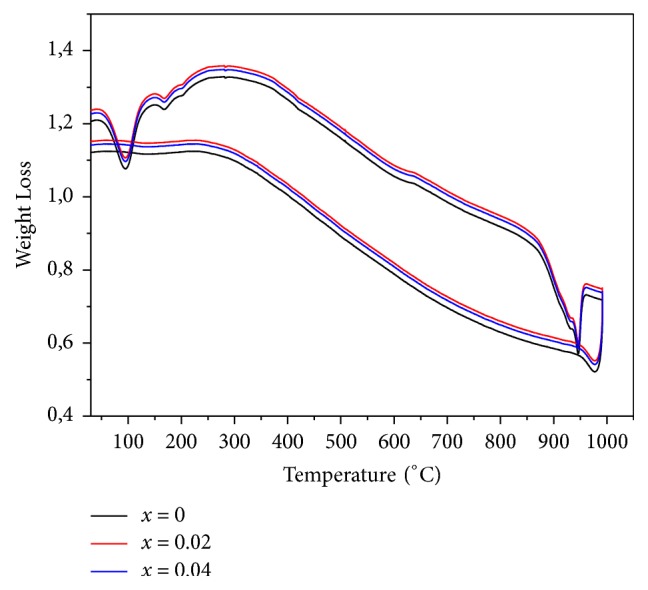
DTA of LiGd_(1-x)_Eu_x_(PO_3_)_4_ (x=0, 0.02 and 0.04).

The thermal stability of lithium polyphosphate is investigated using DTA. The curves of the Eu: LiGd(PO_3_)_4_ crystal are given in Figure 6. It is clearly observed that the curves present the same shape (evolution). Indeed, a single sharp endothermic peak is observed between 900 and 1000°C for all samples, which exhibits the characteristics of a first-order phase transition. This signal can be attributed to the decomposition of polyphosphates to GdPO_4_. The stability of lithium gadolinium polyphosphates can be explained by heavily distorted of PO_4_ tetrahedra as are the GdO_8_ polyhedra. We thus conclude that all compounds are stable at high temperatures and it is monophasic.

### 3.5. Magnetic Study

**Figure 7:**
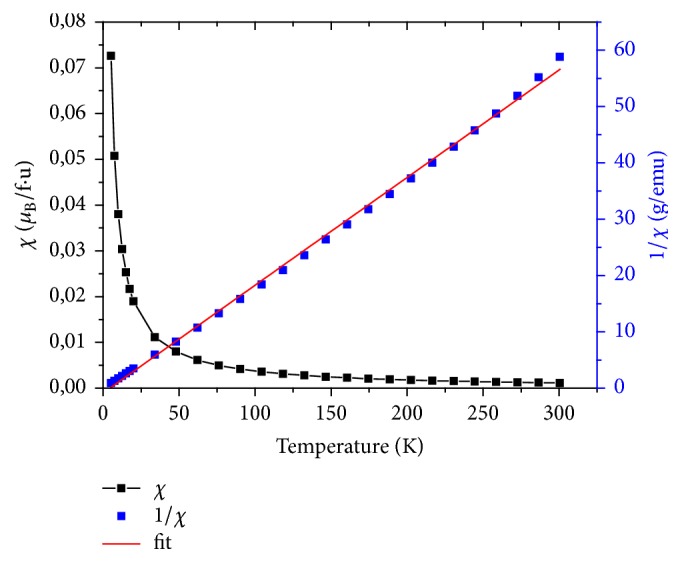
The magnetic susceptibility (*χ*) and inverse magnetic susceptibility (1/*χ*) measurements as a function of temperature of LiGd(PO_3_)_4_.

**Figure 8:**
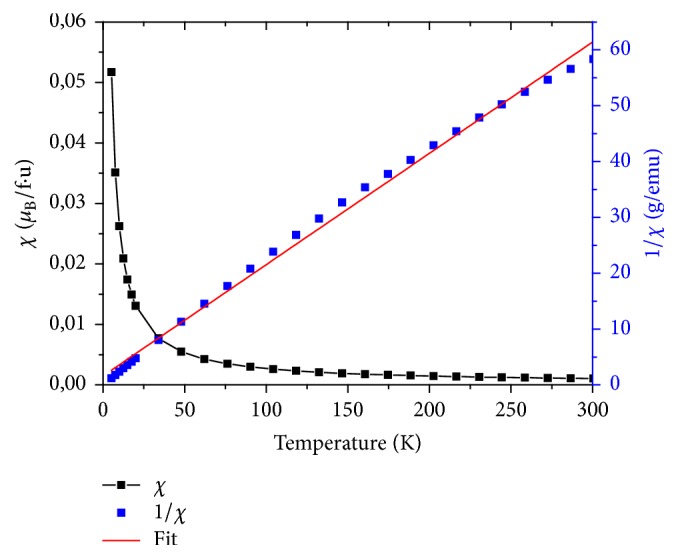
The magnetic susceptibility (*χ*) and inverse magnetic susceptibility (1/*χ*) measurements as a function temperature of LiGd_0.98_Eu_0.02_(PO_3_)_4_.

**Figure 9:**
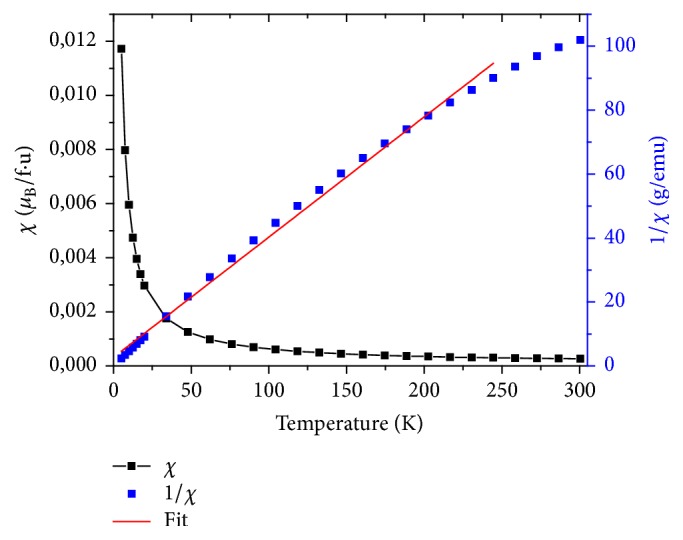
The magnetic susceptibility (*χ*) and inverse magnetic susceptibility (1/*χ*) measurements as a function temperature of LiGd_0.96_Eu_0.04_(PO_3_)_4_.

The magnetic susceptibility and inverse magnetic susceptibility versus temperature of LiGd(PO_3_)_4_, LiGd_0.98_Eu_0.02_(PO_3_)_4_, and LiGd_0.96_Eu_0.04_(PO_3_)_4_ are shown in Figures 7, 8, and 9, respectively. The only other reported type of rare earth polyphosphate structures are those of LGP: Eu^3+^; and these were chosen because Gd^3*+*^ has an effective magnetic moment and the 4f^n^ electrons.

These curves prove that all three rare earth polyphosphate compounds exhibit a paramagnetic response. The nondoped LGP is the most paramagnetic one; this is explained by their structural stability. Indeed, the addition of europium in the host disturbs samples in crystallinity. The response for LGP obeys Curie's Law very well; this is consistent with the (^8^S_7/2_) ground state of Gd^3*+*^, which has no orbital angular momentum and so is unaffected by crystal field effects. Fitting, the temperature dependence of the inverse of susceptibility *χ*^−1^ in high temperatures is given by the formula [35]

(3) $$\begin{array}{c} \frac{1}{\chi }=\frac{\left(T-\theta_{p}\right)}{C} \end{array}$$

where *θ*_P_ is the Weiss temperature and C is the Curie constant given by

(4) $$\begin{array}{c} C\approx \frac{\mu_{0}N\mu_{eff}^{2}}{3K_{B}} \end{array}$$

where N is the number of carriers of magnetic moment, *μ*_0_ is the vacuum permeability, K_B_ is the Boltzmann constant, *μ*_B_ is the Bohr magnetron, and *μ*_eff_ is effective moment of carriers. Samples' structure consists of one magnetic species (i), possessing each a magnetic moment *μ*_eff_ (i); the magnetic susceptibility is given by the relation:

(5) $$\begin{array}{c} \chi =\mu_{0}\frac{n_{1}\mu_{eff}^{2}\left(1\right)+n_{2}\mu_{eff}^{2}\left(2\right)+\cdots +n_{i}\mu_{eff}^{2}\left(i\right)}{3K_{B}T} \end{array}$$

Generally, the magnetic moment is determined by

(6) $$\begin{array}{c} \mu_{eff}=g_{J}\sqrt{J\left(J+1\right)} \end{array}$$

where g_J_ is the Lande factor and J is the total angular moment. The theoretical effective paramagnetic moment *μ*_eff_^the^ for the samples can be calculated by

(7) $$\begin{array}{c} {{µ}_{eff}^{2}}^{the}\;=\left\{\;xg_{Gd^{3+}}^{2}J_{Gd^{3+}}\left(\;J_{Gd^{3+}}+1\;\right)\;\right\}{µ}_{B}^{2} \end{array}$$

**Figure 10:**
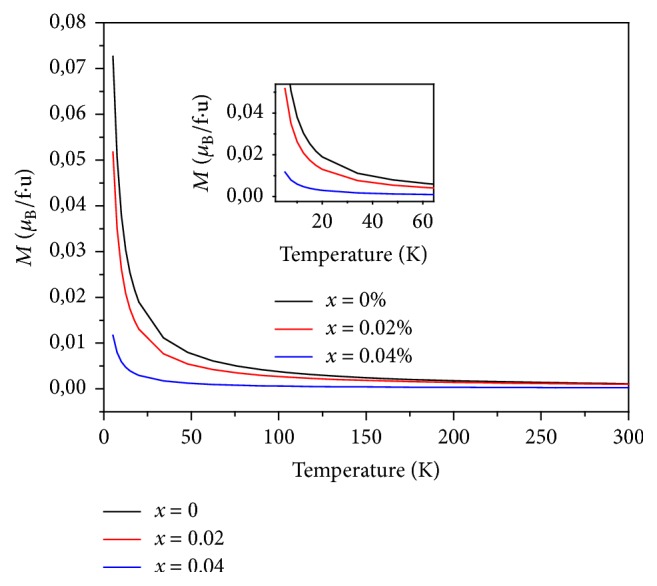
Magnetic measurements of LiGd_(1-x)_Eu_x_(PO_3_)_4_ (x=0, 0.02 and 0.04).

Curves of *χ*^−1^ versus temperature allow deducing *μ*_eff_^exp^ values, which are summarized in Table 5. We can notice that the values of *μ*_eff_^the^ decrease with the decrease of Gd percentage in the system, due to the important magnetic moment of Gd^3+^ ions (7.94*μ*_B_). The comparison between the theoretical and the experimental effective moment values shows that the former are higher than the latter. This result can be associated with the increase of disorder in the matrix (LGP). On the other side, when the temperature increases to more than 75K, it induces a thermal agitation and causes magnetic moments disorientation of atoms in Eu doped LGP polyphosphates. Consequently, a decrease of paramagnetism is clearly observed (Figure 10).

### 3.6. Luminescence Properties

#### 3.6.1. Excitation

**Figure 11:**
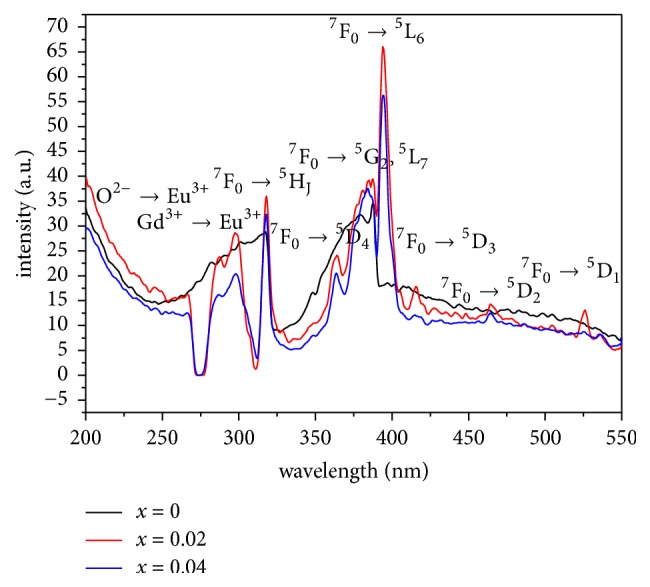
Excitation spectra with *λ*_em_=591 nm of LiGd_(1-x)_Eu_x_(PO_3_)_4_ (x=0, 0.02 and 0.04) at 300 K.

Excitation spectra of LiGd(PO_3_)_4_, doped with europium (2, 4%) (Figure 11), are measured at 300K under emission with *λ*_em_= 591 nm. Figure 11 shows broad band from 254 to 271 nm. These bands are assigned to the charge transfer bands (CTB), resulting from the transfer of an electron from the orbital 2p^6^ of the ligand O^2-^ to the empty state of the configuration [Xe]4f^6^ of the Eu^3+^ ion (Eu^3+^-O^2-^ transition). The maximum of the CTB is located at 245 nm. The differences between broadening and positions of the maxima intensities of the CTB in polyphosphate indicate their dependence on the host lattices [36]. This is due to the strong binding of the oxygen ligands in the polyphosphate compound [37].

At low frequencies, several groups of narrow bands in the spectral region 271-310 nm are clearly observed and assigned to ^8^S_7/2_→^6^H_J_, ^8^S_7/2_→^6^G_J_, ^8^S_7/2_→_ _^6^D_J_, ^8^S_7/2_→^6^I_J_, and ^8^S_7/2_→^6^P_J_transitions of the Gd^3+^ ion. LiYF_4_:Gd^3+^ crystal is used as reference to identify excitation bands, which describe the basis of the detailed energy level scheme proposed for the trivalent gadolinium [38]. The presence of band in the range between 271 and 310 nm indicates the presence of energy transfer between the two rare earths, which occurs from Gd^3+^ to Eu^3+^ in the matrix. However, there is no CTB of Eu^3+^–O^2-^ or energy transfer band Gd^3+^-Eu^3+^ above 310 nm. Excitation spectra within the wavelength range of 310–550 nm, show only the intrinsic transitions 4f-4f from the ground state ^7^F_0_ to different excited levels (^5^D, or ^5^L) of Eu^3+^ ion. These transitions are assigned as follows: ^7^F_0_→^5^H_J_ at 316 nm, ^7^F_0_→^5^D_4_ at 362 nm, ^7^F_0_→^5^G_2_, ^5^L_7_ at 382 nm,^ 7^F_0_→^5^L_6_ at 393 nm,^ 7^F_0_→^5^D_3_ at 417 nm, ^7^F_0_→^5^D_2_ at 464 nm,and ^7^F_0_→^5^D_1_ at 502 nm. All these assignments and wavelengths are given in Table 6. Most of the excitation bands are broadened and some of them overlap together to form a strong band, particularly the band between 369 and 409 nm with FWHM of about 18 nm.

The perfect match of this excitation band with the emission wavelength of NUV In GaN-based LED chips makes these phosphors conveniently useful in white LEDs [39]. Figure 11 shows that the band intensities increase with europium concentration. However, they maintain the same shape and position.

#### 3.6.2. Emission

**Figure 12:**
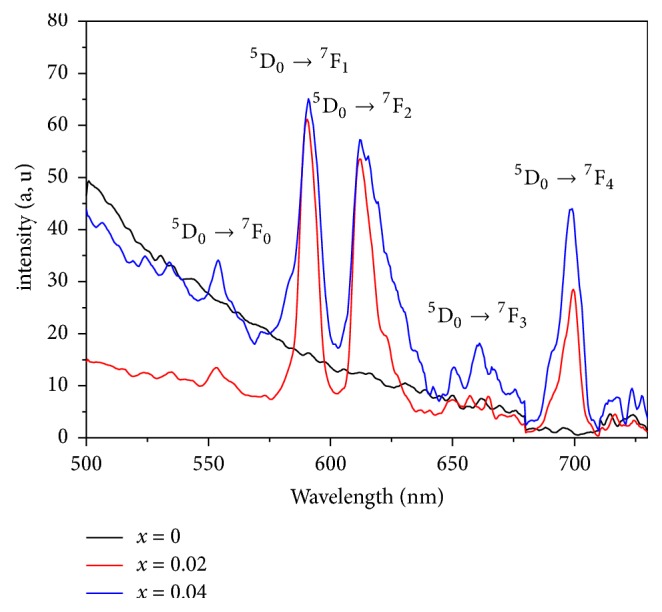
Emission spectra with *λ*_ex_=394 nm of LiGd_(1-x)_Eu_x_(PO_3_)_4_ (x=0, 0.02 and 0.04) at 300 K.

The emission spectra of condensed phosphates are recorded at ambient temperature (300K) and in the range of 500-750 nm after excitation with *λ*_ex_= 394 nm (Figure 12). These spectra present the same shapes, with bands intensity proportional to Eu^3+^ active ion concentration. However, we notice that the undoped LiGd(PO_3_)_4_ polyphosphate does not emit light. The observed emission bands are attributed to the following transitions: ^5^D_0_→^7^F_J_ (where J = 0,1,2,3 or 4) of Eu^3+^ ion in the matrix LiGd(PO_3_)_4_ [40, 41].

Figure 12 proves the presence of five bands in the emission spectra where the most intense ones are those situated at 578-600 nm (^5^D_0_→^7^F_1_) and 604-634 nm (^5^D_0_→^7^F_2_). The other emission bands are observed at 554 nm (^5^D_0_→^7^F_0_), 660 nm (^5^D_0_→^7^F_3_), and 686-706 nm (^5^D_0_→^7^F_4_). The corresponding assignments and wavelengths of these emissions are given in Table 7. The relative intensities of the most intense transitions ^5^D_0_ →^7^F_0_, ^7^F_1_, ^7^F_2_  ^7^F_3_ and ^7^F_4_ are strongly influenced by the nature of the host and the crystalline environment [42]. Therefore, the dominance of magnetic dipole (MD) transition ^5^D_0_→^7^F_1_ of Eu^3+^ means that Eu^3+^ occupies a site in the crystal lattice with inversion symmetry. However, in the case of absence of symmetry inversion in the site of Eu^3+^, the main emission would be the electric dipole (ED) transition ^5^D_0_→^7^F_2_ [43]. The synthesized polyphosphates showed that orange emission transition (^5^D_0_→^7^F_1_) is slightly dominated. This indicates that Eu^3+^ occupies a site in the crystal lattice with symmetry inversion.

## 4. Conclusion

Polyphosphates of rare earth and alkali metal LGP:Eu^3+^ were successfully synthesized by solid-state reaction at 730°C. XRD patterns proved that the obtained samples crystallize in a monoclinic single phase with space group I 2/a and following cell parameters a= 9.635(3) Å, b= 7.035(3) Å, c= 13.191(3) Å, *β*= 90.082°, V= 894.214 Å^3^, and Z= 4. The synthesized polyphosphates showed a good thermal stability until 940°C. Spectroscopic analyses by IR and Raman spectra confirmed the acyclic zig-zag chain of (PO_4_)^3–^ in LGP structure, involving GdO_8_ dodecahedra and LiO_4_ polyhedra. The magnetic susceptibility carried out on single crystals revealed that the title compounds were paramagnetic between 5 and 300 K. An increase in excitation and emission bands intensities was observed with the increase of europium concentration. The presence of band in the range between 271 and 310 nm in excitation spectra proved the energy transfer process from Gd^3+^ to Eu^3+^. The dominance of ^5^D_0_→^7^F_1_ transition in the emission spectra confirms that Eu^3+^ occupies a site in the crystal lattice with symmetry inversion. The change in transition bands intensity proves that LGP phosphates affect europium environment.

## Figures and Tables

**Table 1:** Refined structure parameters from powder X-ray Rietveld analysis for LiGd(PO_3_)_4_ in space group I 2/a.

Atom	Wyck	x	y	z	B	Occ

Gd	4e	0.75000	0.2982(3)	0.00000	1.35(8)	0.50

Li	4e	0.75000	0.79603(4)	0.00000	1.88(16)	0.50

P1	8f	0.48189(3)	0.06204(4)	0.143135(20)	1.88(16)	1.00

P2	8f	0.54601(3)	0.66399(4)	0.15354(2)	1.88(16)	1.00

OL12	8f	0.5665(6)	0.87995(6)	0.1598(14)	2.7(2)	1.00

OL21	8f	0.4057(4)	0.0815(16)	0.2447(3)	2.7(2)	1.00

OE21	8f	0.6520(11)	0.59342(6)	0.0754(8)	2.7(2)	1.00

OE11	8f	0.5743(13)	0.2282(14)	0.1098(11)	2.7(2)	1.00

OE22	8f	0.3953(6)	0.626(2)	0.1220(12)	2.7(2)	1.00

OE12	8f	0.3696(12)	0.0106(3)	0.0652(10)	2.7(2)	1.00

	*R*_p_ = 1.237	*R*_wp_= 1.598	*R*_exp_ = 1.134	*R*_Bragg_= 4.301	*χ* ^2^= 2	

**Table 2:** Atomic distances(Å) and angles in LiGd(PO_3_)_4_ with standard deviations in parentheses.

**Tetrahedra **	**aroundP1**				

P1-OL12	1.534(4)	OL12 -OL21	2.381(12)	OL12_ _^i^ —P1—OE11	111.61(38)

P1-OL21	1.535(4)	OL12 -OE21	2.446(11)	OL12_ _^i^ —P1—OL21	101.76(23)

P1-OE11	1.534(11)	OL12 -OL21	2.463(14)	OL12_ _^i^ —P1—OE12	105.86(29)

P1-OE12	1.535(12)	OL12 -OE11	2.538(11)	OL21—P1—OE11	117.46(53)

		OL12 -OE22	2.482(12)	OL21—P1—OE12	105.55(40)

		OL12 -OE12	2.448(15)	OE12—P1—OE11	113.37(47)

**Tetrahedra **	**around P2**				

P2 -OL12	1.5343(13)	OE22 -OL12	2.482(12)	OE22—P2—OE21	113.09(39)

P2 -OL21	1.534(6)	OE22 -OL21	2.619(12)	OE22—P2—OL12	108.04(41)

P2 -OE21	1.535(10)	OE22 -OE21	2.560	OE22—P2—OL21_ _^vi^	117.27(36)

P2 -OE22	1.533(7)	OL21 -OL12	2.381(12)	OE21—P2—OL12	105.66(4)

		OL21 -OL12	2.440(11)	OE21—P2—OL21_ _^vi^	105.25(16)

		OE21 -OE22	2.560(13)	OL12—P2—OL21_ _^vi^	106.79(38)

**Polyhedra **	**around Gd**	**Tetrahedra** **around Li**			

Gd -OE21	2.489(6)	Li -OE21		Gd -Gd_ _^iv^	5.592(2)

Gd -OE21	2.489(6)	Li -OE21		Li -Gd_ _^ii^	3.533(2)

Gd -OE11	2.283(13)	Li -OE12		Li -Li_ _^v^	5.068(0)

Gd -OE11	2.283(13)	Li -OE12		P1 - P2	2.8710(4)

Gd-OE22	2.197(13)			P2 -P1	2.7897(4)

Gd-OE22	2.197(13)			Gd -P1_ _^iii^	6.256(2)

Gd-OE12	2.605(7)				

Gd -OE12	2.605(7)				

**Symmetry** **code**	i: x,1+y,z	ii:x,1+y,z iii:1.5-x,1+y,-z		iv:2-x,1-y,-z v:2-x,2-y,-z	vi:1-x,0.5+y,0.5-z

**Table 3:** The size of the crystallite according to percentage of europium.

Percentages (%)	FWHM	Position(2*θ*)	D(nm)

0	0.189	20.630	42.49

2	0.189	20.628	42.79

4	0.189	20.621	42.79

**Table 4:** Attributions of main IR bands (cm^−1^) of LiGd_(1-x)_Eu_x_(PO_3_)_4_ samples.

Assignment	x=0	x=0.02	x= 0.04

*υ*_as_ OPO	1257	1241	1250
	1137	1127	1149

*υ*_s_ OPO	1089	1089	109
*υ*_as_ POP	1014	1014	1014
	944	944	947

*υ*_s_ POP	689	689	689
	736	736	736
	761	758	761
	818	818	818

*δ*POP	437-	437	437
*δ*POP	456	456	457
	519	519	521
	586	585	585

**Table 5:** Values of C, *μ*_eff_^the^ (*μ*_B_), and *μ*_eff_^exp^ (*μ*_B_) for the LiGd_(1-x)_Eu_x_(PO_3_)_4_ (x=0, 0.02 and 0.04) compounds.

	x=0	x=0.02	x=0.04

C (*μ*_B_.KT^−1^)	5.26	5	2.86

*μ*_eff_^the^ (*μ*_B_)	7.94	7.86	7.78

*μ*_eff_^exp^ (*μ*_B_)	6.50	6.32	4.65

**Table 6:** Excitation lines attribution of Eu^3+^ doped LiGd(PO_3_)_4_.

Wavelength (nm)	Attribution

287	^7^F_0_→^5^I_6_

294	^7^F_0_→^5^F_4_

297	^7^F_0_→^5^F_2_

6318	^7^F_0_→^5^H_6_

321	^7^F_0_→^5^H_4_

328	^7^F_0_→^5^H_7_

363	^7^F_0_→^5^D_4_

376	^7^F_1_→^5^D_4_

373-390	^7^F_0_→^5^G_J(2,4)_

394	^7^F_0_→^5^L_6_

405	^7^F_1_→^5^L_6_

416	^7^F_0_→^5^D_3_

464	^7^F_0_→^5^D_2_

526	^7^F_0_→^5^D_1_

**Table 7:** Emission attribution of Eu^3+^ doped LiGd(PO_3_)_4_.

Transitions	Wavelengths (nm)

^5^D_0_→^7^F_0_	554

^5^D_0_→^7^F_1_	578-601

^5^D_0_→^7^F_2_	604-634

^5^D_0_→^7^F_3_	660

^5^D_0_→^7^F_4_	686-706
